# Effects of Maté Tea Intake on *ex Vivo* LDL Peroxidation Induced by Three Different Pathways

**DOI:** 10.3390/nu1010018

**Published:** 2009-06-29

**Authors:** Ruth Lobato T. Matsumoto, Simone Mendonça, Daniela Moura de Oliveira, Marina F. Souza, Deborah H. Markowicz Bastos

**Affiliations:** 1 Nutrition Department, School of Public Health, University of São Paulo. Av Dr Arnaldo, 715, 01246-904. São Paulo - SP, Brazil; Email: ruth.matsumoto@gmail.com (R.L.T.M.); danimoura@usp.br(D.M.O.); marinasousa@usp.br(M.F.S.); 2 Empresa Brasileira de Pesquisa Agropecuária, AGROENERGIA. Parque Estação Biológica - PqEB s/n Asa Norte 70770-901 EMBRAPA - Brasilia- DF, Brazil; Email: doutorasimone@gmail.com

**Keywords:** antioxidant, phenolic compounds, *Ilex paraguariensis*, lipid peroxidation, LDL, ApoB

## Abstract

Yerba maté (*Ilex paraguariensis*) is a native South America plant widely consumed as different beverages. Yerba maté leaves contains high concentrations of polyphenols that are responsible for its high *in vitro* and *in vivo *antioxidant activity. The *in vivo* antioxidant properties *vis* a *vis* LDL particles has not yet been studied for maté tea, the roasted yerba maté product. The aim of this study was to evaluate the antioxidant activity of maté tea ingestion *ex vivo* on human LDL. Fasting peripheral venous blood samples of healthy women were taken in three different times: before drinking the tea, one hour later and after one week (7 days) of daily consumption of maté tea. The isolated LDL was oxidized by three different pathways [copper (CuSO_4_), lipoxygenase and peroxynitrite (SIN-1)]. Conjugated dienes and structural modifications on LDL were evaluated. Ingestion of maté tea increased LDL resistance towards *ex vivo* copper oxidation, but did not alter the peroxidation pattern when SIN-1 or lipoxygenase were used as oxidants

## 1. Introduction

Oxidized LDL is considered to be an important factor in the atherogenic progression of coronary artery disease (CAD). Different lipid oxidation products have been identified in lesions and plasma of patients with vascular disease. For most lipid oxidation products found *in vivo*, the exact mechanisms for their generation are unknown and may involve multiple pathways [1], including oxygen and nitrogen free radical chain reactions, pro-oxidant action of metal ions (Fe^3+^, Cu^2+^) and cell lipoxygenase peroxidation activity [1,2,3], among others.

Simultaneous production of nitric oxide (^•^NO) and superoxide anion (O_2_^−^) by vascular cells may explain peroxynitrite (ONOO−) formation within the vascular wall. Peroxynitrite-modified LDL binds to scavenger receptors, leading to accumulation of cholesteryl esters involved in the production of atherosclerotic lesions [4]. Lipoxygenase is an enzyme that incorporates one molecule of oxygen into unsaturated fatty acids in a regiospecific and stereospecific manner. In mammalian tissues, there are four lipoxygenase isoenzymes, named according to the position of oxygenation in arachidonic acid. Lipoxygenases oxygenate not only free fatty acids, but also esterified fatty acids such as phospholipids, cholesteryl esters and cholesteryl ester in LDL [2]. Transition-metals are powerful initiators of lipid peroxidation, which may be available in free form in animal and human atherosclerotic lesions [5]. 

The oxidation process generates a huge variety of carbonyl compounds, such as malondialdehyde (MDA), 4-hydroxynonenal (4-HNE), acrolein and glyoxal. These compounds may directly modify LDL and may also react with proteins, thereby impairing their biological activity [2,6]. Different lipid oxidation products are identified in lesions and plasma of patients with vascular disease.

Carbonyl scavenging agents, i.e., substances capable of reducing the accumulation of advanced lipoperoxidation end products, are of potential interest because they may reduce or retard oxidative damage [7]. Dietary polyphenols play a major role in maintaining the antioxidant defense of organisms that are exposed to several free radical generating factors, since they are able to scavenge such chemical species [8]. Leaves from yerba maté (*Ilex paraguariensis*) are a potential source of polyphenols such as phenolic acids (chlorogenic and caffeic acids), also present in hot infusions made from either the dried green leaves (“chimarrão”) or the roasted leaves (maté tea) [9]. In addition to polyphenols yerba maté contains caffeine and saponins [10]. The antioxidant activity of green yerba maté beverages has already been described in both *in vitro* and *in vivo* models [10,11]. There is a lack of studies on maté tea (the roasted product from yerba maté) in human models.

The aim of this study was to evaluate the antioxidant activity of maté tea on LDL from young female volunteers after acute and prolonged maté tea intake (one week). We used three different pathways for LDL oxidation, mimicking those that occur in the arterial wall: transition metal, peroxynitrite and lipoxygenase, in accordance with Gugliucci and Menini [12].

## 2. Results and Discussion

**Figure 1:**
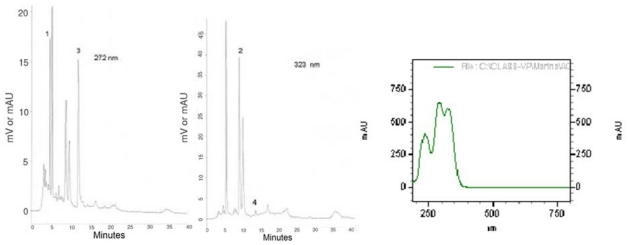
HPLC bioactive compounds profile of the instant maté extract at 272 nm and at 323 nm and UV espectra of caffeoylquinic acids. 1 = Theobromine, 2 = 5-CQA, 3 = Caffeine, 4 = Caffeic Acid.

In order to characterize the plant material used in this study, we present the chromatographic profiles of maté tea at 272 and 323 nm (Figure 1), the absorption spectra of caffeoylquinic acids as well as chlorogenic acids and xanthines contents (Table 1). 

**Table 1:** Phytochemicals from maté tea (mg/g).

Species	Values (mg/g)*^a^*
**Caffeine**	10.20 ± 0.09
**Theobromine**	4.38 ± 0.05
**5-Caffeoylquinic acid (5-CQA)**	42.17 ± 0.50
**Caffeic acid**	0.63 ± 0.03
**Phenolic acids (HPLC)*^b^***	280.88 ± 22.83
**Total polyphenol (Folin-Ciocalteau)**	377.77 ± 3.65

*^a^* Values expressed as mean ± SD *^b^* Determined from each chromatographic peak tentatively identified as chlorogenic acid, based in the UV spectra comparison with that of pure 5-CQA

The baseline lipid profile and body mass index (BMI) for all subjects were within normal values (Table 2). Analysis of the three-day dietary food record at baseline (first week) and after the maté tea supplementation week showed good overall compliance with the recommended diets. There were no differences regarding daily nutrient intake before and after the supplementation period (p > 0.05) (Table 3).

**Table 2:** Anthropometric characteristics and fasting blood lipids of subjects in the baseline.

Variable	Values*^a^*
**Nº of individuals**	5
**Age (y)**	27.20 ± 3.27
**BMI (kg/m^2^)**	21.29 ± 2.05
**Total cholesterol (mmol/L)**	4.85±0.60
**LDL cholesterol (mmol/L)**	2.65±0.26
**HDL cholesterol (mmol/L)**	1.54±0.40
**Triacylglycerols (mmol/L)**	0.91±0.15

*^a^* Values expressed as mean ± SD (n=5)

**Table 3:** Diet composition of the subjects in the baseline and during the supplementation week*.

Food components	Day 8 (Baseline)	Day 15 (supplementation week)	Sig. (p)^*a*^
**Energy (Kcal)**	1633.9 ± 230.8	1470.8 ± 446.5	0.657
**Carbohydrate (%)**	57.9 ± 6.5	57.7 ± 2.8	0.946
**Protein (%)**	17.5 ± 2.04	15.6 ± 2.6	0.179
**Lipid (%)**	24.6 ± 6.8	26.8 ± 3	0.428
**Saturated fatty acids (g)**	14.04 ± 6.8	11.35 ± 4.10	0.311
**Monounsaturated fatty acids (g)**	11.9 ± 4.4	10.6 ± 4.2	0.061
**Polyunsaturated fatty acids (g)**	7.0 ± 0.6	5.0 ± 1.3	0.657

^*^ Values expressed as mean ± SD (n=5) ^a^ Paired t-test

Maté tea intake did not significantly affect conjugate diene formation when SIN-1 or lipoxygenase were used, either at T_1_ or T_2_. Nonetheless, we observed a nonsignificant decrease in the maximal rate of conjugated diene formation when SIN-1 was used. In contrast, there was a significant decrease in the susceptibility of LDL to oxidation after daily maté tea intake (T_2_), compared with baseline (T_0_), when peroxidation was induced with copper (Table 4).

**Table 4:** LDL maximal rate of conjugated diene formation (Δabs/Δtime) after oxidation induced *ex vivo*, at the baseline (T_0_) and after acute (one hour -T_1_) and prolonged (one week -T_2_) ingestion of instant maté tea employing three different oxidation pathways*.

Period	Cu++ *^a^*	Lypoxigenase *^a^*	SIN-1 *^a^*
**T_0_**	3.1 ± 0.7	1.4 ± 0.2	8.2 ± 3.8
**One hour (T_1_)**	2.8 ± 0.4	1.4 ± 0.2	6.7 ± 2.4
**One week (T_2_)**	2.0 ± 0.1^**^	1.0 ± 0.4	7.7 ± 0.9

^a^ Values were multiplied by 1,000 ^*^ Values expressed as mean ± SD (n = 5). Analysis were made in duplicate ^**^ P < 0.05 compared to T_0_

**Figure 2:**
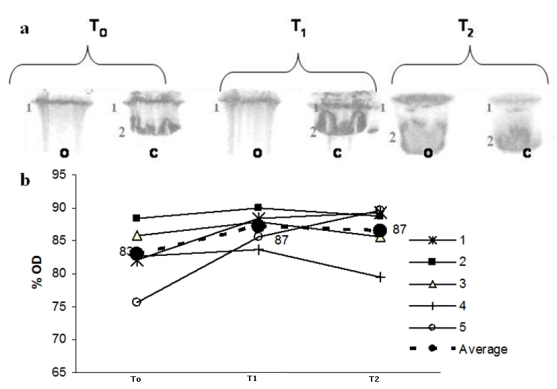
(a) LDL SDS-PAGE profile before C and after peroxidation O with 10 mmol/L Cu^++ ^at three different times: before maté tea ingestion (T_0_); after 1h maté tea ingestion (T_1_) and after 1 week maté tea ingestion (T_2_). Lane 2 represents native LDL Apo B (500KDa ApoB). (b) Optical density % variation during the experiment related to Lane 2 (o) in PAGE. Lines (1-5) correspond to Lane 2 OD % from each volunteer (dotted line correspond to the average value).

Figure 2 shows the SDS-PAGE profile for Apo B, in which lane 2 represents native LDL Apo B. The oxidation of Apo B, which results in high molecular weight aggregates that do not penetrate the separating gel, leads to modification in the protein eletrophoretic behaviour. After peroxidation, there is a marked decrease in lane 2 intensity, observed in the eletrophoresis gel and followed by the decrease in the optical density (% OD). Maté tea intake inhibited this effect at T_2_ but not at T_1 _(Figure 2 a,b).

In order to evaluate whether acute and prolonged intake of maté tea would protect LDL from peroxidation, and to investigate the possible mechanisms, we used three different peroxidation pathways, as described by Gugliucci and Menini [11] and recommended by Halliweel [4]. 

We did not observe any significant decrease in susceptibility to LDL oxidation when SIN-1 or lipoxygenase were the peroxidation agents after acute (T_1_) or prolonged (T_2_) intake of maté tea (Table 4). On the other hand, maté tea intake for one week significantly decreased LDL susceptibility to copper oxidation (T_2_), while no significant effect was observed one hour after maté tea consumption. In a previous study, a dose-dependent increase in LDL protection was observed when Cu^++^, SIN-1 and lipoxygenase were used as oxidants [13], after the ingestion of the green yerba maté beverage “chimarrão”. The fact that polyphenol content decreases and considerable degradation of caffeoylquinic esters occurs during the roasting process [10], and that the abovementioned study evaluated reactive thiobarbituric substances, rather than diene conjugate formation, might explain the discrepancies in the results.

LDL peroxidation is a slow process that occurs in the sub-intimal space, by contact between LDL and reactive species. The oxidation process generates a huge variety of lipid peroxidation products that can react with lysine residues in Apo B, with several implications relating to the atherogenesis process [6]. The effects of LDL Apo B modification can be followed by SDS-PAGE. Modifications in Apo B include cross-linking along the protein chain, with the formation of aggregates that will not enter the electrophoresis gel [13,14,15,16,17,18,19,20,21]. We observed that the consumption of maté tea for one week increased the protection of LDL Apo B against peroxidation with copper (Figure 2). There was a noticeable increase in optical density (OD) for lane T_2 _after oxidation, which corresponds to the native Apo B state (Figure 2a), despite individual differences (Figure 2b). This result is in agreement with the findings of Gugliucci and Menini [11] and Gugliucci [22]. Other medicinal plants, traditionally used in China, like *Scolopendra subspinipes*, have shown a similar effect *in vitro* [23].

*Ilex paraguariensis* infusions contain a large amount (approximately 30%) of polyphenols. About 3% of the weight of yerba maté leaves are in the form of caffeoylquinic acids and its isomers, generically named as chlorogenic acids (CGA), esters from caffeic acid and quinic acid [9,24]. Maté tea, besides the aforementioned chlorogenic acids, monocaffeoylquinic acid (CQA) and dicaffeoylquinic acid (diCQA), dicaffeoylshikimic acid and caffeoylshikimic acid are also found [10]. However, due to the roasting process, phenolic compounds can be lost while compounds with antioxidant properties are generated by the Maillard reaction [9]. Most notably of mate’s biological activities is its high antioxidant capacity, which has been shown to be higher than green tea [9]. This high antioxidant capacity is attributed and is directly proportional to its chlorogenic acid and total phenolic acids contents [25]. Studies with coffee, an important food source of CGAs, have shown that these hydrophilic compounds are absorbed by the body and can be detected in human plasma within 0.5 to 4 hours after consumption [19]. 

Owing to an *ortho*-dihydroxy phenyl ring, CGAs are both efficient free radical scavenger and metal ion chelators, mechanisms by which these compounds protect LDL against metal-catalyzed oxidation [26]. Furthermore, CGAs may: increase the resistance of LDL to oxidative modification as a result of their incorporation into LDL [27]; regenerate alfa-tocopherol through the reduction of the α-tocopheroxyl radical, and thus protecting biological structures in non polar environments [28,29]. These different mechanisms of action may support the hypothesis that polyphenols from maté tea and/or their metabolites may diffuse into the arterial wall and enhance antioxidant capacity in the aqueous phase, possibly on the surface of the lipoprotein particles, thereby inhibiting Cu^2+^-induced LDL oxidation [20,22].

Besides substantial amounts of phenolic acids, the leaves of maté also contain caffeine and saponins [10,24]. These bioactive compounds are recognized to influence the central nervous system [30] and exert anti-inflammatory activities [31]. However, the potential biological relevance of antioxidant properties of these compounds is yet uncertain [32]. Maté tea has been shown to affect plasma oxidative stress parameters as well as the level of leukocyte antioxidant enzyme gene expression in young women [33] and to decrease fatty acid peroxidation in mice liver [34], indicating that polyphenols from maté tea promote protection against peroxidation by several mechanisms in different biological structures. There is considerable evidence indicating that polyphenols present in fruits and vegetables have antioxidant activity *in vitro*. 

We hypothesize that prolonged maté tea intake may protect LDL, because of the ability of phenolic acids to inhibit copper-induced LDL autoxidation, rather than through scavenging nitrogen free radical species or inhibiting lipoxygenase activity, in the model used in this study [35]. *In vivo *studies aiming to evaluate cellular and molecular targets are helpful for elucidating whether and how these phytochemicals are effective as dietary coadjutants in health maintenance. Although the number of volunteers in this study was small, each individual was her own control and a similar trend could be observed for the results, despite individual differences. Polyphenols from maté tea were bioavailable to protect LDL against *ex-vivo* copper oxidation, as already observed for green maté “chimarrão”. 

In conclusion, we observed that prolonged maté tea consumption prevented LDL oxidation, expressed as diene conjugate formation, and prevented LDL Apo B structural changes when copper is the oxidative agent, but not in the presence of other pro-oxidants such as peroxynitrites and lipoxygenase. Results from previous experiments showed that green maté “chimarrão” was able to prevent LDL peroxidation. This study showed that maté tea presents similar *in vivo* antioxidant capacity, despite the roasting process. 

## 3. Experimental

### 3.1. General

All chemicals were of analytical grade and purchased from Sigma Aldrich (St Louis, MO, USA). All the instant maté tea used in this study was from the same batch and was obtained from Leão Jr, Curitiba, PR, Brazil.

### 3.2. Maté Tea Preparation

The tea beverage was prepared by dissolving 5 g of commercial instant yerba maté in 500 mL of fresh mineral water (which is equivalent to the soluble solids content in “chimarrão”). 

### 3.3. Total Phenol Concentration

The total phenol concentration was measured using the Folin-Ciocalteau methodology, as described elsewhere [13]. Chlorogenic acid (Sigma) was used as a standard and total polyphenol concentration was expressed as equivalents of chlorogenic acid per g of instant maté tea.

### 3.4. Chromatographic Analysis by High-Performnce Liquid Chromatography (HPLC)

The instant maté tea was analyzed with no modification other than the appropriate dilution in the mobile phase to fit the standard curves. A TSP (Thermo Separation Products) high-performance liquid chromatograph equipped with a Spectra Series Gradient Pump, an automatic Spectra Series Autosampler and a UV/VIS Spectra System detector was used for the assays. All the modules were controlled by a personal computer equipped with the HPLC System Manager SN4000 (Spectra System). A 4.6 × 250 mm, 5 μm C18 Microsorb column was used for the separation. The injection volume was 20 μL. The analytical determination was carried out by means of HPLC using two-solvent isocratic elution [14]. The composition of the solvents was: (A) water/acetic acid (99.5:0.5 v/v) and (B) methanol. The mobile phase composition was 75% of solvent A and 25% of solvent B. The flow rate was 0.8 mL/min. Data were obtained at 272 nm for caffeine and theobromine and 323 nm for phenolic acids. The identification of phenolic compounds and methylxanthines was based on comparison of the spectra obtained between 250 and 350 nm and the retention time of the unknown substances in relation to that of pure standards. Quantification was achieved by external calibration, using a five-point curve of different dilutions of a standard solution. Pearson’s correlation coefficient (r) was always > 0.99. Analyses were performed in triplicate.

### 3.5. In vivo Study

Fasting blood samples from five healthy normolipidemic volunteers was obtained by venipuncture and collected in evacuated tubes with EDTA (5 mmol/L). The blood was immediately centrifuged at 1,500 g for 15 min at 4 °C. The volunteers were recruited by means of an advertisement at the School of Public Health, University of São Paulo, which invited them to participate in the study. The inclusion criteria were that the volunteers needed to be women aged 18-35 years, with BMI < 30 and an omnivorous diet, who were not using vitamins or herbal supplements and not undergoing any treatment for chronic diseases. The exclusion criteria were that they should not be smokers and should not present anemia, pregnancy, lactation or chronic diseases such as diabetes, cardiovascular disease or hypothyroidism. All of these criteria were ascertained prior to enrolling the subjects in the study. BMI was calculated as the body weight (kg) divided by the height (m) squared. Serum cholesterol, HDL cholesterol and triacylglycerols were assayed using enzymatic colorimetric methods (Roche Diagnostics, Mannheim, Germany), and LDL cholesterol was calculated using the Friedewald formula. Informed consent was obtained from each volunteer before the investigation, and the study was carried out in accordance with the guidelines of the Ethics Committee of School of Public Health, University of São Paulo. 

### 3.6. Study Design

The subjects were instructed to discontinue their use of coffee, tea, red wine, cocoa, chocolate and sodas one week prior to the first blood collection, and to avoid the use of spirits and analgesics three days before the visits for blood collection. Consumption of fruit juices was not to exceed 300 mL per day. On the eighth day of the dietary restriction, after 8-10 hours of overnight fasting, an intravenous catheter was inserted into the antecubital vein and a baseline blood sample was obtained (T_0_). Maté tea was prepared as described above and offered to each subject. Blood was drawn one hour after maté tea consumption (T_1_). Then, all the subjects were instructed to consume 5 g of instant maté tea, diluted in 500 mL of cold water, once a day, for seven days, and to maintain the dietary restrictions described above. On the fifteenth day, after 8-10 hours of overnight fasting, blood samples were again drawn (T_2_).

The nutrient composition of the subjects’ diets was calculated from three-day dietary records (two days during the week and one day during the weekend). The intake over the study period was calculated as the mean value of the three-day dietary records evaluated both during the first week of the study (baseline) and during the second week (supplementation period). The food records were checked by a nutritionist, in order to counsel the volunteers, and these records were then analyzed using the Nutwin software version 2.0 (UNIFESP; Federal University of São Paulo, SP, Brazil). 

### 3.7. LDL Preparation

LDL (d = 1.019-1.063 g/mL) was isolated from the volunteers’ plasma by sequential flotation ultracentrifugation at 4 ºC [15]. After extensive dialysis, LDL was kept in 10 mmol/L sodium phosphate buffer (pH 7.4) containing 150 mmol/L NaCl and 0.1 mmol/L EDTA, at 4 ºC, and was used within one week. Prior to the oxidation experiments, LDL was dialyzed overnight against the same buffer without EDTA. The LDL protein concentration was determined using a total Protein Assay Kit (Labtest). 

### 3.8. LDL Oxidation

Three different systems for LDL oxidation were used, as described by Gugliucci and Menini [13]. Copper (CuSO_4_), SIN-1 and lipoxygenase were used as peroxidation agents. The SIN-1 reagent releases ONOO- in the same way as under physiological conditions, thereby generating a continuous source of lipid radicals and favoring propagation reactions [1,5]. Briefly, one milliliter of LDL adjusted to 100 μg protein/mL with the appropriate buffer was incubated: a) in the presence of 1 mmol/L of freshly prepared SIN-1 in 50 mmol/L of phosphate buffer (pH 7.4); b) in the presence of 5 μmol/L of CuSO_4_ diluted in 0.02 mmol/L of phosphate buffer (pH 7.4); and c) in the presence of 200 U/mL of soybean lipoxygenase EC1.13.11.12, diluted in borate buffer (pH 9.0). The incubations were carried out at 37 °C for four hours for the diene conjugate analysis and for 16 hours for the evaluation of changes to LDL Apo B by means of SDS-PAGE.

### 3.9. Lipid Peroxidation Evaluation

Lipid peroxidation was evaluated according to diene conjugate generation. The latter was monitored as described elsewhere [16,17]. Briefly, the differential absorbance at 234 nm was continuously monitored and the absorbance value was registered at five-minute intervals for three hours. The maximum rate of conjugated diene formation (MCD) (Δabsorbance/Δtime elapsed in minutes between the initiation phase and the maximum absorbance phase) was determined. This analysis was performed in duplicate.

### 3.10. Evaluation of LDL Apo B Aggregation by Means of SDS-PAGE

To check for the possible protective effect of maté tea on LDL Apo B aggregation due to copper LDL oxidation, electrophoresis was performed on the LDL after peroxidation with CuSO_4_. Peroxidation was performed as described previously. After 16 hours, the oxidation was halted by adding butylhydroxytoluene (BHT) (40 μmol/L) and EDTA (5 mmol/L) to the reaction medium. The samples were immediately cooled in ice and kept at -20°C until electrophoresis. SDS-PAGE was carried out on 10% polyacrylamide gel, using a Hoefer miniVE system (Amersham Biosciences, Uppsala, Sweden) [18]. Each lane was loaded with 30 μg of protein. The gels were stained with Coomassie Blue R 0.025% in 40% methanol and 7% acetic acid and then decolored with the methanol-acidified solution. The Apo B structural modifications induced by copper peroxidation were evaluated by comparing the optical density from the 500 KDa native Apo B SDS-PAGE band with the bands present in the oxidized LDL, at baseline (T_0_), after one hour (T_1_) and after one week (T_2_) of maté tea intake, using the Ultraquant - 6.0 Image Acquisition and Analysis Software (Claremont, CA, USA). 

### 3.11. Statistical Analysis

Data were expressed as the mean value ± standard deviation (SD). Baseline (T_0_) and post-treatment parameters (T_1_ - one hour; and T_2_ - one week) were compared using the paired Student t-test. P values < 0.05 were considered to be statistically significant.

## Acknowledgments

The authors want to express their gratitude to FAPESP (Fundação de Amparo à Pesquisa do Estado de São Paulo) for the financial support (#06/58019-5 and #07/51952-0), to Leão Jr (Curitiba, Paraná) for providing the instant maté tea and for the grant-in-aid to author Matsumoto.
